# Internalized weight stigma and psychological distress mediate the association of perceived weight stigma with food addiction among young adults: A cross-sectional study

**DOI:** 10.1186/s40337-024-01112-x

**Published:** 2024-09-30

**Authors:** Po-Ching Huang, Janet D. Latner, Nadia Bevan, Mark D. Griffiths, Jung-Sheng Chen, Chi Hsien Huang, Kerry S. O’Brien, Chung-Ying Lin

**Affiliations:** 1 School of Physical Therapy, Graduate Institute of Rehabilitation Science, College of Medicine, Chang Gung University, 259 Wen-Hwa 1st Rd, Taoyuan, 333323 Taiwan; 2 Department of Psychology, University of Hawaii at Manoa, 2500 Campus Road, Honolulu, HI 96822 USA; 3 School of Social Sciences, Monash University, 20 Chancellors Walk, Clayton, VIC 3800 Australia; 4 International Gaming Research Unit, Psychology Department, Nottingham Trent University, 50 Shakespeare St, Nottingham, NG1 4FQ UK; 5 Department of Medical Research, E-Da Hospital, I-Shou University, 1 Yida Rd., Yanchao Dist, Kaohsiung, 82445 Taiwan; 6 Department of Family Medicine and Community Medicine, E-Da Hospital, I-Shou University, No. 1, Yida Rd., Jiaosu Village, Yanchao District, Kaohsiung City, 82445 Taiwan; 7 College of Medicine, I-Shou University, No.8, Yida Rd., Jiaosu Village, Yanchao District, Kaohsiung City, 82445 Taiwan; 8 Institute of Allied Health Sciences, College of Medicine, National Cheng Kung University, 1 University Rd., East Dist, Tainan, 701401 Taiwan; 9 Biostatistics Consulting Center, College of Medicine, National Cheng Kung University Hospital, National Cheng Kung University, 1 University Rd., East Dist, Tainan, 701401 Taiwan; 10 Department of Public Health, College of Medicine, National Cheng Kung University, 1 University Rd., East Dist, Tainan, 701401 Taiwan; 11 Department of Occupational Therapy, College of Medicine, National Cheng Kung University, 1 University Rd., East Dist, Tainan, 701401 Taiwan

**Keywords:** Food addiction, Psychological distress, Weight stigma, Young adults, Taiwan

## Abstract

**Background:**

Perceived weight stigma (PWS) and internalized weight stigma (IWS) are both associated with psychological distress and food addiction (FA). Using the previously proposed ‘cyclic obesity/weight-based stigma’ (COBWEBS) model, the present study extended the framework to investigate the mediating effects of IWS and psychological distress in the association between PWS and FA among young adults. Given that individuals who are overweight/have obesity have different vulnerabilities, this population was separately analyzed alongside the total study population.

**Methods:**

An online survey comprising the Perceived Weight Stigma Scale, Weight Bias Internalization Scale (WBIS), Depression, Anxiety and Stress Scale-21 (DASS-21), and modified Yale Food Addiction Scale Version 2 was completed by 601 participants (59.6% females; mean age 29.3 years [SD = 6.07]). A total of 219 participants were categorized as being overweight/having obesity.

**Results:**

A direct correlation was found between PWS and FA (standardized coefficient [β] = 0.28, *p* < 0.001) among both populations, and was mediated by IWS and psychological distress (β [95% CI] = 0.03 [0.01, 0.05] for WBIS score and 0.10 [0.06, 0.14] for DASS-21 score) among the total participants, but only mediated by psychological distress among participants who were overweight/had obesity (β [95% CI] = 0.14 [0.06, 0.24]).

**Conclusions:**

The results demonstrated novel perspectives by showing the direct association between PWS and FA and the mediating roles of IWS and psychological distress. Treatment strategies such as psychological acceptance and psychoeducation could be used to reduce weight stigma, which could have positive downstream benefits of ameliorating FA. Future research may seek to study strategies for reducing weight stigma and psychological distress, to investigate their efficacy in improving disordered eating.

## Abstract (plain language summary)

The present study proposed a model to understand the association between weight stigma and food addiction with the following considerations: (i) subdividing weight stigma into perceived weight stigma and internalized weight stigma; and (ii) adding psychological distress in the association between weight stigma and food addiction. Analysis was carried out on two populations of young adults: the total sample, and participants who were overweight/had obesity. The results supported the direct association between perceived weight stigma and food addiction among both samples. This association was mediated by internalized weight stigma and psychological distress among the total participants, but only mediated by psychological distress among participants who were overweight/had obesity. The findings provided novel evidence regarding the effect of perceived weight stigma, as well as the mediating roles of internalized weight stigma and psychological distress in the development of food addiction. Such findings might be implemented into strategies aimed at reducing food addiction.

## Introduction

Weight stigma is defined as the prejudice and discrimination derived from devaluation regarding an individual’s weight status [1, 2], but individuals who ‘perceive’ themselves as being overweight or obese can be harmed by weight stigma [3], irrespective of their actual weight [4]. In other words, individuals may still feel stigmatized despite having normal weight. The perceived “thin ideal” has dominated the desired aesthetic for decades [5]. With the increasing exposure to the thin ideal of beauty (e.g., from social media) [6], individuals may tend to internalize the thin ideal and develop misperceptions about their weight, subsequently comparing themselves with others [4, 7]. This has been reported to be associated with body dissatisfaction [5] and weight stigma [7], and is a predictor for disordered eating [8–11].

Weight stigma can take two forms: perceived weight stigma (PWS), the experience of stigma perpetuated by others, and internalized weight stigma (IWS), the negative attitudes, beliefs, judgments, stereotypes, and discriminatory acts directed at the self. The experience of PWS is frequent among individuals who ‘perceive’ themselves to be overweight [1, 2]. IWS commonly occurs once individuals believe societal messages regarding weight stigma apply to themselves and provoking self-devaluation [1, 12, 13]. Weight stigma may influence individuals’ psychological health [1, 14], causing psychological disturbances such as depression, anxiety or loneliness [15, 16]. In addition, weight stigma may also increase the risk of adverse health conditions including maladaptive eating behaviors [9, 16, 17], addictive behaviors [18] and low physical activity [16, 19].

The ‘cyclic obesity/weight-based stigma’ (COBWEBS) model proposed by Tomiyama [20] illustrates the vicious cycle of weight stigma. In the COBWEBS model, the stress experienced from weight stigma/obesity may increase individuals’ eating, consequently resulting in weight gain and further exacerbating weight stigma/obesity. However, weight stigma in the COBWEBS model is viewed as a whole without considering the context of different types of stigmatization (i.e., PWS and IWS). Considering that PWS and IWS have been reported to be different constructs [12], the present study extended the COBWEBS model by investigating the relationship between two different types of weight stigma (i.e., PWS and IWS), psychological distress, and eating behavior. More specifically, the present study extends the COBWEBS model in two ways. First, it subdivides weight stigma into PWS and IWS. Second, the present study proposes an additional direct association between PWS and eating behavior into the framework of the COBWEBS model.

One form of maladaptive eating behavior, food addiction (FA), has been reported to be associated with weight stigma [1, 9, 17, 21, 22]. FA refers to the constant obsession with consuming highly palatable food despite knowing that the disadvantages outweigh the advantages [1]. Ultra-processed food with high sugar, salt, fat, or the combinations of these ingredients are considered addictive [23], and its consumption is recognized as a rewarding mechanism in the brain because it activates addictive-like responses to stimulate brain reward circuits that provide pleasure and relief from internal discomfort [24]. Therefore, stigmatized individuals may consume these addictive foods as a way to cope with stress and escape from reality when facing psychological distress, subsequently increasing the likelihood of developing FA and obesity [1].

Despite FA not being formally recognized in the latest (fifth) edition of the *Diagnostic and Statistical Manual for Mental Disorders* (DSM-5), researchers have adapted the criteria listed in the DSM-5 for substance use disorder to develop 12 FA symptoms using 11 diagnostic criteria to identify FA. The 12 symptoms comprise: (i) substance consumption (excessive food intake beyond what was intended); (ii) persistent desire (inability to reduce or stop food consumption); (iii) time expenditure (significant time spent consuming food); (iv) activity reduction (abandoning important activities due to food consumption); (v) awareness of consequences (continued consumption despite knowing physical or emotional repercussions); (vi) tolerance (gradual increase in food intake over time); (vii) withdrawal (experiencing withdrawal symptoms when unable to eat desired foods); (viii) social problems (social or interpersonal issues resulting from food consumption); (ix) role failure (inability to meet role obligations due to food consumption); (x) hazardous situations (engaging in food consumption that leads to physically dangerous situations); (xi) craving (intense desire to consume specific foods); and (xii) significant impairment (experiencing considerable distress or impairment due to food consumption) [25].

Young adults – a population with frequent use of social media – may experience elevated exposure to unhealthy thin-ideal images [6] and may engage in greater appearance comparison between peers [26]. In addition, individuals in transition from adolescence to young adulthood are developing wider interpersonal relationships, which makes them more vulnerable to weight stigmatization [27]. Moreover, considering that individuals who are overweight/have obesity may have different levels of vulnerability and treatability from those with normal weight [28], a subgroup analysis may be needed to better understand such mechanisms. Therefore, the present study aimed to examine the extended COBWEBS model by subdividing weight stigma into PWS and IWS, and examining the direct association between PWS and FA, as well as the mediating effects of IWS and psychological distress. Participants who were overweight/had obesity also underwent separate analysis. It was hypothesized that (i) PWS would directly associate with FA, and both (ii) IWS and (iii) psychological distress would significantly mediate the association between PWS and FA.

## Methods

### Data collection and participants

The present study conducted a cross-sectional survey study with participants recruited through online convenience sampling and snowball sampling. Eligibility criteria included (i) being aged between 20 and 40 years (as the present study aimed to recruit young adults as the target population). Being 20 years of age was the minimum legal age to be seen as an adult in Taiwan at the time when the study was conducted (i.e., participants were required to be at least 20 years old to provide independent consent without the additional consent of parents or guardians); and (ii) the ability to read and comprehend traditional Chinese characters (because the online survey was conducted using traditional Chinese characters). The online survey was set-up in *SurveyMonkey*, and its link was posted on the authors’ social media accounts (e.g., *Facebook* and *Line*) between February and April 2023, and participants were encouraged to distribute the survey link to people they knew (i.e., snowball sampling). An e-consent form was shown on the first page of survey. By clicking “*agree*”, participants indicated their consent and willingness to participate in the study. By clicking “*disagree*”, the study promptly ended. To control the data quality, the survey conducted strategies such as collecting timing and page use data, dummy questions, and consistency checks to avoid invalid data. The study was conducted in accordance with the Declaration of Helsinki and approved by the National Cheng Kung University Human Research Ethics Committee (Approval No.: NCKU HREC-E-110-486-2). Participants who completed the survey received 200 New Taiwan dollars (approximately 6.6 US dollars) as compensation for their time.

### Measures

#### Demographics

The participants reported their demographic information regarding age (in years), gender (male or female), height (in cm), and weight (in kg). Height and weight were then used to calculate body mass index (BMI; kg/m^2^).

#### Perceived Weight Stigma Scale (PWSS)

The PWSS [29, 30] was used to assess the level of weight stigma perceived by participants. The PWSS comprises 10 items rated by dichotomous score (0 = no; 1 = yes). The item scores are summed, and a higher score indicates a higher level of PWS. An example item is “*You are treated with less courtesy than others*.” The Chinese version of the PWSS has demonstrated adequate psychometric properties in previous research [21] and had very good internal consistency in the present study (Cronbach’s α = 0.85).

#### Weight Bias Internalization Scale (WBIS)

The WBIS [31] was used to assess the level of IWS. The WBIS comprises 11 items rated on a five-point Likert-like scale (1 = completely disagree; 5 = completely agree). The item scores are summed, and a higher score indicates a higher internalization of weight stigma. An example item is “*I feel ashamed of my body because of my weight.*” The Chinese version of the WBIS has demonstrated good psychometric properties in previous research [32] and had excellent internal consistency in the present study (Cronbach’s α = 0.90).

#### Depression, Anxiety and Stress Scale (DASS-21)

The DASS-21 [33] was used to assess the level of psychological distress. The DASS-21 comprises 21 items rated on a four-point Likert-like scale (0 = never; 3 = almost always). Item scores are summed to generate a total score ranging from 0 to 63 [33]. An example item is “*I found it difficult to relax.*” The Chinese version of the DASS-21 has demonstrated good psychometric properties in previous research [34, 35] and had excellent internal consistency in the present study (Cronbach’s α = 0.95).

#### Modified Yale Food Addiction Scale version 2 (mYFAS 2.0)

The mYFAS 2.0 [36] was used to assess the severity of FA. The mYFAS 2.0 comprises 13 items rated on an eight-point Likert-like scale (0 = never; 7 = every day). The scale adopts a unique scoring method [36] where 13 items, including 11 diagnostic criteria and two clinical impairments, are converted into symptoms on a 0–1 dichotomous scale (0 indicates *non-endorsed*; 1 indicates *endorsed*). Individuals who endorse two or more symptoms and at least one clinical impairment are deemed to have met the criteria for FA. In addition, according to the cutoff of each question, the 11 diagnostic criteria are summed to derive a total score ranging from 0 to 11. Hereafter the term FA indicates individuals presenting with FA, and the term mYFAS 2.0 score indicates the scores rated on the mYFAS 2.0. An example FA item is “*I ate to the point where I felt physically ill*.” The Chinese version of the mYFAS 2.0 has demonstrated good psychometric properties in previous research [25, 37] and had excellent internal consistency in the present study (Cronbach’s α = 0.93).

### Data analysis

Participants who were overweight/had obesity were differentiated from those were not overweight by using a BMI of over 24 kg/m^2^ according to criteria proposed by the Ministry of Health and Welfare in Taiwan [38]. Participants’ demographic information and scores on each measure were analyzed using descriptive statistics, including n (%) and mean (SD). Pearson correlations were used to examine the bivariate associations between scores on the PWSS, WBIS, DASS-21, and mYFAS 2.0. Hierarchical regression models and logistic regression models were constructed to examine how basic demographics (age, gender with male as reference group, and BMI), and scores on the PWSS, WBIS, and DASS-21 were associated with mYFAS 2.0 score and FA. Both regressions were conducted in three steps: the first step included basic demographic information, the second step included additional PWSS scores, and the third step included additional WBIS and DASS-21 scores. The variance inflation factor (VIF) was calculated in the hierarchical regression model to ensure that there were no substantial issues with collinearity. Lastly, mediation models were constructed where mYFAS 2.0 score was the dependent variable, PWSS scores was the independent variable, WBIS and DASS-21 scores were the mediating variables, and demographics (i.e., age, gender, and BMI) were the controlled variables. The mediation model was examined using the Hayes’ Process Macro (Model 4) with 5000 bootstrapping resamples. The mediated effects of IWS (i.e., WBIS score) and psychological distress (i.e., DASS-21 score) in the association of PWS (i.e., PWSS score) with FA (i.e., mYFAS 2.0 score) were examined using the 95% bootstrapping confidence interval (CI). That is, when the 95% bootstrapping CI does not cover 0, the mediating effect is significant [39].

## Results

Table 1 shows the baseline characteristics. There were 601 participants (59.6% female, mean age = 29.3 years; SD = 6.1) in total, with 62 classed as having FA (10.3%) and mean scores of 1.29 out of 10 for PWSS (SD = 2.16), 29.25 out of 55 for WBIS (SD = 8.08), 13.00 out of 63 for DASS-21 (SD = 11.56), and 0.89 out of 11 for mYFAS 2.0 (SD = 1.88). Among 601 participants, 219 were categorized as overweight/having obesity (46.6% female, mean age = 30.7 years; SD = 6.1) with 24 of them classed as having FA (11.4% among participants who were overweight/had obesity) and mean scores of 1.85 out of 10 for PWSS (SD = 2.53), 33.23 out of 55 for WBIS (SD = 7.41), 13.94 out of 63 for DASS-21 (SD = 11.50), and 1.04 out of 11 for mYFAS 2.0 (SD = 2.04). Table 2 shows the correlations between the study variables. For both populations, all the variables were significantly correlated with each other (standardized coefficient [β] = 0.25 to 0.45, all *p*-values < 0.001).

**Table 1:** Participants’ characteristics

	Total participants (*N* = 601)	Participants with BMI > 24 kg/m^2^ (*n* = 219)
	n (%) or Mean (SD)	
Gender		
Male	243 (40.43)	117 (53.4)
Female	358 (59.57)	102 (46.6)
Age (in years)	29.3 (6.07)	30.7 (6.10)
Height (cm)	166.00 (8.31)	167.86 (8.42)
Weight (kg)	64.3 (13.97)	77.46 (12.17)
Body mass index (kg/m^2^)	23.20 (4.02)	27.42 (3.24)
Food addiction (yes)	62 (10.30)	24 (11.40)
PWSS score (possible range: 0–10)	1.29 (2.16)	1.85 (2.53)
WBIS score (possible range: 11–55)	29.25 (8.08)	33.23 (7.41)
DASS-21 score (possible range: 0–63)	13.00 (11.56)	13.94 (11.50)
mYFAS 2.0 score (possible range: 0–11)	0.89 (1.88)	1.04 (2.04)

PWSS = Perceived Weight Stigma Scale; WBIS = Weight Bias Internalization Scale; DASS-21 = Depression, Anxiety and Stress Scale; mYFAS 2.0 = modified Yale Food Addiction Scale 2.0

**Table 2:** Pearson correlations for the studied variables

		Upper triangular matrix: Participants with BMI > 24 kg/m^2^ (*n* = 219)			
		PWSS	WBIS	DASS-21	mYFAS 2.0
Lower triangular matrix: Total participants (*N* = 601)	PWSS	--	0.453 (< 0.001)	0.393 (< 0.001)	0.392 (< 0.001)
	WBIS	0.397 (< 0.001)	--	0.450 (< 0.001)	0.252 (< 0.001)
	DASS-21	0.399 (< 0.001)	0.362 (< 0.001)	--	0.444 (< 0.001)
	mYFAS 2.0	0.396 (< 0.001)	0.264 (< 0.001)	0.380 (< 0.001)	--

BMI = Body mass index; PWSS = Perceived Weight Stigma Scale; WBIS = Weight Bias Internalization Scale; DASS-21 = Depression, Anxiety and Stress Scale; mYFAS 2.0 = modified Yale Food Addiction Scale 2.0

Table 3 shows the hierarchical regression model among the total sample (*N* = 601) to explain mYFAS 2.0 score. Age was significant for mYFAS 2.0 score (β = 0.13, *p* = 0.001) and remained significant after Step 2 variables had been added (β = 0.10, *p* = 0.009 in Step 2, and 0.09, *p* = 0.014 in Step 3). The PWSS score added in the second step significantly explained the mYFAS 2.0 score (β = 0.41, *p* < 0.001) and remained significant after the Step 3 variables added (β = 0.28, *p* < 0.001). Lastly, WBIS and DASS-21 scores significantly explained the mYFAS 2.0 score (β = 0.10 and 0.23, respectively, *p* = 0.033 and *p* < 0.001).

**Table 3:** Results of hierarchical regressions in explaining food addiction scores among total participants to (*N* = 601)

		B (se) / β (*p*)	
	Step 1	Step 2	Step 3
Age	**0.04 (0.01) / 0.13 (0.001)**	**0.03 (0.01) / 0.10 (0.01)**	**0.03 (0.01) / 0.09 (0.01)**
Gender (Ref: male)	-0.14 (0.16) / -0.04 (0.39)	**-0.34 (0.15) / -0.09 (0.02)**	**-0.34 (0.14) / -0.09 (0.02)**
BMI	0.03 (0.02) / 0.06 (0.18)	-0.03 (0.02) / -0.06 (0.15)	**-0.04 (0.02) / -0.08 (0.06)**
PWSS	--	**0.35 (0.03) / 0.41 (< 0.001)**	**0.24 (0.04) / 0.28 (< 0.001)**
WBIS	--	--	**0.02 (0.01) / 0.10 (0.03)**
DASS-21	--	--	**0.04 (0.01) / 0.23 (< 0.001)**
R^2^ (adjusted R^2^)	0.027 (0.022)	0.176 (0.171)	0.237 (0.230)
F change (*p*-value)	5.47 (0.001)	31.84 (< 0.001)	30.83 (< 0.001)
VIF	1.044–1.087	1.051–1.175	1.056–1.547

BMI = Body Mass Index; PWSS = Perceived Weight Stigma Scale; WBIS = Weight Bias Internalization Scale; DASS-21 = Depression, Anxiety and Stress Scale

B = unstandardized coefficient; se = standard error; β = standardized coefficient; VIF = variation inflation factor. Significance is shown in **bold**

Table 4 shows the hierarchical regression model using participants who were overweight/had obesity (*n* = 219) to explain mYFAS 2.0 scores. Age was significant for mYFAS 2.0 score (β = 0.17, *p* = 0.011) in Step 1. After adding Step 2 variables, only PWSS score significantly explained the mYFAS 2.0 score (β = 0.40, *p* < 0.001) and remained significant after Step 3 variables were added (β = 0.26, *p* < 0.001). Lastly, after adding WBIS and DASS-21 scores in Step 3, only DASS-21 scores significantly explained the mYFAS 2.0 score (β = 0.34, *p* < 0.001).

**Table 4:** Results of hierarchical regressions in explaining food addiction scores among participants who were overweight/had obesity (*n* = 219)

		B (se) / β (*p*)	
	Step 1	Step 2	Step 3
Age	**0.06 (0.02) / 0.17 (0.01)**	0.04 (0.02) / 0.11 (0.10)	0.04 (0.02) / 0.11 (0.06)
Gender (Ref: male)	-0.10 (0.28) / -0.02 (0.72)	-0.35 (0.26) / -0.09 (0.18)	-0.31 (0.25) / -0.08 (0.22)
BMI	0.03 (0.04) / 0.05 (0.44)	-0.03 (0.04) / -0.04 (0.49)	-0.02 (0.04) / -0.03 (0.63)
PWSS	--	**0.32 (0.05) / 0.40 (< 0.001)**	**0.21 (0.06) / 0.26 (< 0.001)**
WBIS	--	--	-0.001 (0.02) / -0.004 (0.95)
DASS-21	--	--	**0.06 (0.01) / 0.34 (< 0.001)**
R^2^ (adjusted R^2^)	0.035 (0.022)	0.175 (0.159)	0.274 (0.253)
F change (*p*-value)	2.619 (0.052)	36.166 (< 0.001)	14.415 (< 0.001)
VIF	1.007–1.013	1.033–1.130	1.047–1.519

BMI = Body Mass Index; PWSS = Perceived Weight Stigma Scale; WBIS = Weight Bias Internalization Scale; DASS-21 = Depression, Anxiety and Stress Scale

B = unstandardized coefficient; se = standard error; β = standardized coefficient; VIF = variation inflation factor. Significance is shown in **bold**

Table 5 shows the logistic regression using total participants to explain FA (*N* = 601). After adding the demographic variables in Step 1, only BMI was significant (adjusted odds ratio [aOR] = 1.07, 95% confidence interval [95% CI] = 1.00, 1.13). PWSS score was added in Step 2, and both gender (aOR = 0.55. 95% CI = 0.30, 0.997) and PWSS score (aOR = 1.46. 95% CI = 1.31, 1.62) were significant and remained significant after adding Step 3 variables (aOR = 0.50. 95% CI = 0.27, 0.92 for gender; and aOR = 1.30. 95% CI = 1.15, 1.46 for PWSS). Lastly, both WBIS score (aOR = 1.07. 95% CI = 1.03, 1.13) and DASS-21 score (aOR = 1.03. 95% CI = 1.00, 1.05) significantly explained FA after being added into the model.

**Table 5:** Results of logistic regressions in explaining food addiction among total participants (*N* = 601)

		Adjusted odds ratio (95% CI)	
	Step 1	Step 2	Step 3
Age	1.05 (0.999, 1.09)	1.03 (0.98, 1.08)	1.03 (0.98, 1.08)
Gender (Ref: male)	0.77 (0.45, 1.33)	**0.55 (0.30**,** 0.997)**	**0.50 (0.27**,** 0.92)**
BMI	**1.07 (1.00**,** 1.13)**	1.00 (0.93, 1.07)	0.96 (0.88, 1.04)
PWSS	--	**1.46 (1.31**,** 1.62)**	**1.30 (1.15**,** 1.46)**
WBIS	--	--	**1.07 (1.03**,** 1.13)**
DASS-21	--	--	**1.03 (1.00**,** 1.05)**

BMI = Body Mass Index; PWSS = Perceived Weight Stigma Scale; WBIS = Weight Bias Internalization Scale; DASS-21 = Depression, Anxiety and Stress Scale

CI = confidence interval. Significance is shown in **bold**

Table 6 shows the logistic regression using participants who were overweight/had obesity to explain FA (*N* = 219). After adding the demographic variables in Step 1, only BMI was significant (aOR = 1.13, 95% CI = 1.01, 1.25). PWSS score was added in Step 2 and was significant (aOR = 1.46, 95% CI = 1.24, 1.72), and remained significant after Step 3 variables were added (aOR = 1.36. 95% CI = 1.12, 1.64). Lastly, only DASS-21 scores (aOR = 1.06. 95% CI = 1.01, 1.10) significantly explained FA after WBIS and DASS-21 scores were added.

**Table 6:** Results of logistic regressions in explaining food addiction among participants who were overweight/had obesity (*n* = 219)

		Adjusted odds ratio (95% CI)	
	Step 1	Step 2	Step 3
Age	1.05 (0.98, 1.13)	1.02 (0.94, 1.10)	1.02 (0.94, 1.10)
Gender (Ref: male)	1.11 (0.47, 2.61)	0.81 (0.32, 2.07)	0.86 (0.31, 2.37)
BMI	**1.13 (1.01**,** 1.25)**	1.06 (0.94, 1.18)	1.06 (0.94, 1.19)
PWSS	--	**1.46 (1.24**,** 1.72)**	**1.36 (1.12**,** 1.64)**
WBIS	--	--	1.01 (0.93, 1.09)
DASS-21	--	--	**1.06 (1.01**,** 1.10)**

BMI = Body Mass Index; PWSS = Perceived Weight Stigma Scale; WBIS = Weight Bias Internalization Scale; DASS-21 = Depression, Anxiety and Stress Scale

CI = confidence interval. Significance is shown in **bold**

**Fig. 1:**
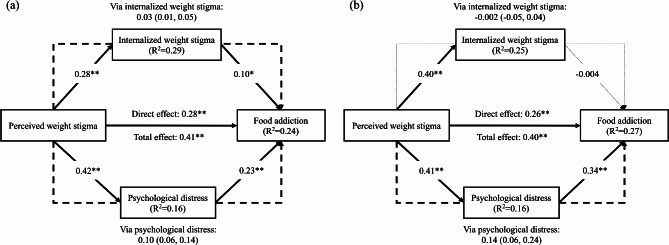
Results of the proposed mediation model on food addiction using (**a**) total participants (*N* = 601) and (**b**) participants who were overweight/had obesity (*n* = 219) Notes. Age, gender, and body mass index were controlled variables. Dashed lines indicate indirect effects via a specific mediator (i.e., internalized weight stigma or psychological distress) with values of standardized coefficient and 95% bootstrapping confidence interval in parentheses. Solid lines indicate direct or total effects with values of standardized coefficients. Significances are shown in thick lines *p < 0.05; **p < 0.001

Figure 1 demonstrates the mediation model, with 1a conducted among the total sample (*N* = 601) and 1b conducted using participants who were overweight/had obesity (*n* = 219). The results concur with the findings of the hierarchical regression models and logistical regression model. In brief, among total participants (Fig. 1a), PWS directly correlated with FA (β = 0.28, *p* < 0.001), controlling for age, gender and BMI. The mediation effects of IWS and psychological distress supported the association between PWS and FA. The unstandardized coefficient (95% bootstrapping CI), 0.03 (0.01, 0.05) for indirect effect via IWS, and 0.10 (0.06, 0.14) for indirect effect via psychological distress. Among participants who were overweight/had obesity (Fig. 1b), PWS also directly correlated with FA (β = 0.26, *p* < 0.001), after controlling for age, gender and BMI. Only psychological distress (unstandardized coefficient [95% bootstrapping CI] = 0.14 [0.06, 0.24]) had a mediating effect in supporting the association between PWS and FA.

## Discussion

The present study examined the association and mediating effect of IWS and psychological distress in the relationship between PWS and FA among Taiwanese young adults. Participants who were overweight/had obesity were separately analysed. The prevalence of FA was 10.3% among the total population and 11.4% among those who were overweight/had obesity. The results showed that, among both populations (i.e., total participants and participants who were overweight/had obesity), PWS was directly associated with FA. The association was mediated by IWS and psychological distress among total participants but was only mediated by psychological distress among participants who were overweight/had obesity. Therefore, the study’s three proposed hypotheses were all supported among the total sample (i.e., that [i] PWS would directly associate with FA, and [ii] IWS and [iii] psychological distress would significantly mediate the association between PWS and FA). The hypotheses were partially supported among participants who were overweight/had obesity. The present study is the first to include participants with varied weight and participants who were overweight/had obesity to investigate the association between PWS and FA and the mediating roles of IWS and psychological distress. The findings showed a different mediation effect between two samples, demonstrating the novelty of the results.

The COBWEBS model proposed a sequential relationship whereby weight stigma/obesity may lead to psychological distress, resulting in increased eating and consequent weight gain, subsequently exacerbating weight stigma/obesity and therefore forming a vicious cycle [20]. The study’s findings support the COBWEBS model and additionally extend its construction. That is, weight stigma (more specifically, PWS) had a direct effect with eating behavior (i.e., FA in the present study) in addition to psychological distress. Moreover, IWS and psychological distress both mediated the association between PWS and FA, supporting their significance.

There has been a paucity of evidence reporting the direct effect of PWS compared to IWS [40, 41]. The present study’s findings showed a direct effect between PWS and FA, which may be explained by psychological distress. A study showed that after experiencing weight stigma, participants’ cortisol levels immediately increased if they perceived themselves as overweight, but not among those who considered themselves as average weight [42]. This finding suggests that individuals with higher perceived weight may show a physiological stress response when experiencing prejudiced judgement. Another study investigated the association of perceived stress and vulnerability to visual cues of ultra-processed food and reported that visual elements were more likely to provoke the individuals with higher perceived stress to choose high-calorie food [43]. This supports the findings of the present study as well as the studies reporting an association between PWS and FA [40, 41]. This also explains the association of weight stigma with increased appetite and disordered eating [44, 45].

Additionally, the present study’s findings showed that IWS and psychological distress mediated the association between PWS and FA among individuals with varied weight status, but only psychological distress mediated the association between PWS and FA among individuals who were overweight/had obesity. The mediation effect of psychological distress has previously been reported [46, 47]. As aforementioned, weight stigma is considered a chronic stressor that may provoke both physiological reactions (e.g., increased cortisol level [42], low physical activity [19] and psychological reactions (e.g., loneliness [15], psychological distress [1]). One study reported that there was only a short overlap of time between the increased cortisol level and psychological stress response [42], suggesting that the influence of weight stigma and its negative outcomes may cause a long-term effect that includes both physiological and psychological involvement. Strategies in reducing weight stigma warrant a number of comprehensive approaches which include both physiological and psychological interventions.

IWS has been reported as a mediator among the associations between PWS and several health outcomes, including FA [1, 46] and avoidance of physical activity [48]. The mediating role of IWS may be explained by stress vulnerability factors (e.g., self-esteem or mastery) [15]. In particular, IWS predicted the fear of being stigmatized, which further predicted the exacerbation of addictive-like eating over time [49]. The association between IWS and maladaptive eating was reported to be mediated by weight-related experiential avoidance [50, 51], suggesting the stress caused by IWS prompts individuals to adopt maladaptive coping behavior though a particular avoidance tendency [51]. That is, the attempted dieting may be more stressful for individuals with higher explicit IWS, prompting them to adopt maladaptive eating as a coping strategy to avoid such stress, subsequently resulting in FA.

However, such a mediated effect may only show among individuals with varied weights. The findings from analyzing individuals who were overweight/had obesity found that IWS did not mediate the association between PWS and FA, which is inconsistent with the findings of most previous studies [1, 17]. However, Bidstrup et al. [52] investigated the role of IWS in mediating the association between PWS and several types of disorder eating among participants who sought bariatric surgery. They found that some of the disordered eating behaviors, including external eating, were not mediated by IWS [52]. In addition, Marshall, et al. [51] reported that although IWS had an association with the progression of FA over time, it may not always serve as a straightforward mediator between PWS and FA because other psychological variables (e.g., psychological distress) and contextual factors (e.g., social support) might influence this relationship [51]. Moreover, Carr et al. [53] reported that individuals with obesity were more likely to identify their FA symptoms as distress or impairment, but not individuals with average weight or those who were overweight. This finding supported the difference of FA among individuals with different weight, subsequently influencing the development of FA. Accordingly, the non-significant mediation effect of IWS among individuals who were overweight/had obesity may be attributed to the interaction between IWS and psychological variables. Such interaction may have a contextual difference among individuals with varied weight status.

Individuals with FA have been reported to have difficulties in emotional regulation [54], including challenges in goal-oriented behavior, impulsivity, lack of emotional awareness, limited emotional regulation strategies, and feelings of loneliness [54, 55]. Studies have also indicated that impaired emotional regulation can influence FA in the development of binge-eating disorder [56]. Moreover, individuals with binge-eating disorder have been reported to have insufficient emotional regulation strategies [57]. These findings align with those of the present study, which found that psychological distress significantly mediated the relationship between PWS and FA among both populations. This further supports previous findings which have highlighted the association between maladaptive eating, disordered eating, and FA [57, 58]. Accordingly, cognitive deficits in emotional regulation appear to be a risk factor for developing FA tendencies. Strategies aimed at improving emotional regulation or mindful acceptance may be appropriate interventions for individuals experiencing emotional regulation difficulties.

The current evidence (including the findings of the present study) shows a complex interaction between weight stigma, including its internalization, along with the influence of psychological distress, in resulting FA. The results are consistent with two previous studies investigating the relationship between weight stigma, psychological distress, and eating disturbances including FA. Both supported the sequential effect of IWS and psychological distress in mediating the association between PWS and maladaptive eating [1, 46]. In treatment settings, the negative effects of weight stigma might be addressed through psychoeducation strategies [1]. Psychological distress can be alleviated by interventions such as psychological acceptance [46] or mindfulness [59], and can be particularly implemented among individuals who are overweight/have obesity. Other approaches such as self-monitoring of food consumption [1], emotional regulation skills [54] or cognitive behavioral therapy [55] can be adopted to control the progression of FA.

The present study has several limitations. First, the cross-sectional design did not allow inference of casualty between variables. Second, the self-reported measures might have resulted in recall bias or social desirability bias. Nevertheless, the present study showed novel associations among two types of weight stigma (i.e., PWS and IWS), psychological distress and the proposed influence – FA, and provided new perspectives for the underlying mechanism among individuals with varied weight and those who were overweight/had obesity. Moreover, the present study’s findings demonstrate a medium to large effect size (R² = 0.16–0.29) [60], indicating that the proposed model exhibited a strong and impactful relationship. Future studies should continue to explore the underlying mechanism by investigating the efficacy of treatments targeted at reducing weight self-stigma, psychological distress, and eating disturbances, to reduce FA.

## Conclusion

The present study investigated the mediating effects of IWS and psychological distress in the relationship between PWS and FA among Taiwanese young adults. The results showed that PWS, IWS, and psychological distress were significantly associated with FA. In addition, PWS had a direct effect on FA. IWS and psychological distress both mediated the association. The results provided an extended framework regarding the cycle of weight stigma. Consequently, future interventions should consider targeting each of these variables (i.e., two types of weight stigma and psychological distress) to alleviate their potential influences on FA and other forms of disordered eating.

## Data Availability

The data are available from the corresponding authors upon reasonable request.

## Abbreviations

COBWEBS: Cyclic obesity/weight-based stigma model
DASS-21: Depression, Anxiety and Stress Scale
FA: Food addiction
IWS: Internalized weight stigma
mYFAS 2.0: Modified Yale Food Addiction Scale Version 2
PWS: Perceived weight stigma
PWSS: Perceived Weight Stigma Scale
WBIS: Weight Bias Internalization Scale
